# Sporadic Trichinosis

**Published:** 1899-04-08

**Authors:** 


### SPORADIC TRICHINOSIS.

When, as the consequence of eating partially-cooked trichina-infected meat, a considerable number of persons are attacked with trichinosis, probably almost simultaneously, the recognition of the disease is a matter of no great difficulty. But in sporadic cases, when the diagnosis has to be made from the symptoms rather than from the distribution of the disease, the matter is by no means so simple. Dr. Osler[1] says that many such cases are overlooked, and that although clinically, in hospitals and in private practice, trichinosis is an exceedingly rare disease, yet in post-mortem and anatomical work the number of subjects in which calcified trichinæ are found is by no means inconsiderable; and wherever they are found widely spread throughout the muscles, it is certain that at the time of invasion the patient must have had a very serious illness. Several days elapse after the ingestion of trichinous pork before the larval muscle trichinæ reach their sexual maturity, and two or three days before the female gives birth to the embryos. During this period the patient frequently has severe symptoms—nausea, vomiting, and diarrhœa, with colicky pains in the abdomen. When the trichinæ invade the muscles the symptoms are principally pains in the joints, bones, and muscles, especially in the legs, and general heaviness in the limbs, together with fever, which last has often led to the disease being confused with typhoid fever. There are three points of great value in diagnosis.

1. **The Muscular Pains and Swellings.**—While in typhoid fever it is by no means uncommon to have aching of the limbs and back, it is exceedingly rare to have special complaint of pain in the muscles themselves. When this is pronounced early in a febrile affection it is one of the most suggestive symptoms of the disease; on the other hand, it may be only slight. But in a very severe infection the swelling and hardness of the muscles, the extreme pain on pressure, the position of flexion of the arms and legs, without any redness or swelling of the joints themselves, are almost pathognomonic, while early in typhoid such symptoms are never seen.

2. **Œdema.**—In its most characteristic form this is seen in the eyelids and over the eyebrows, and it is of great value as a symptom because of its early appearance in trichinosis and its absence in typhoid fever. In the hands and feet œdema is more commonly seen late in the disease. Œdema, with slight erythema, above the swollen, tender muscles is very characteristic.

3. **Leucocytosis and Eosinophilia.**—This is a symptom which has been brought out by a careful observation of the blood in Dr. Osler's patients. In the cases observed not only was there considerable leucocytosis, but on a differential count being made the proportion of eosinophiles was found to be largely increased. This may possibly have been due to the coincident polymyositis, but in any case it seems an important symptom. Finally, it must be mentioned that, when suspicion arises, the removal of a portion of the biceps muscle for examination is a simple operation. It should be done with an ordinary scalpel under cocaine.

[1] Amer. Jour. Med. Sci., Mar., 1899.

